# Intranasal perillyl alcohol therapy improves survival of patients with recurrent glioblastoma harboring mutant variant for *MTHFR rs1801133* polymorphism

**DOI:** 10.1186/s12885-020-06802-8

**Published:** 2020-04-07

**Authors:** Giselle M. Faria, Igor D. P. Soares, Marcela D’Alincourt Salazar, Marcia R. Amorim, Bruno L. Pessoa, Clovis O. da Fonseca, Thereza Quirico-Santos

**Affiliations:** 1 Instituto de Biologia, Universidade Federal Fluminense, Niteroi, Rio de Janeiro, ZC 24020-141 Brazil; 2 Programa de Pós-graduação em Neurologia, Faculdade de Medicina, Universidade Federal Fluminense, Niteroi, Rio de Janeiro, 24020-141 Brazil; 3 Programa de Pós-graduação em Ciencia e Biotecnologia, Universidade Federal Fluminense, Niteroi, Rio de Janeiro, 24020-141 Brazil; 4 Department of Hematology and HCT, City of Hope Comprehensive Cancer Center, Duarte, CA 91010 USA; 5 Departamento de Medicina Especializada, Unidade de Pesquisa Clínica (UPC-HUAP), Universidade Federal Fluminense, Niteroi, RJ Brazil

**Keywords:** Perillyl alcohol, Glioblastoma, Methylation, Polymorphism, MTHFR, Epigenetics

## Abstract

**Background:**

Polymorphisms in *MTHFR* gene influence risk and overall survival of patients with brain tumor. Global genomic DNA (gDNA) methylation profile from tumor tissues is replicated in peripheral leukocytes. This study aimed to draw a correlation between *rs1801133 MTHFR* variants, gDNA methylation and overall survival of patients with recurrent glioblastoma (rGBM) under perillyl alcohol (POH) treatment.

**Methods:**

gDNA from whole blood was extracted using a commercially available kit (Axygen) and quantified by spectrophotometry. Global gDNA methylation was determined by ELISA and *rs1801133* polymorphism by PCR-RFLP. Statistical analysis of gDNA methylation profile and *rs1801133* variants included Mann-Whitney, Kruskal-Wallis, Spearman point-biserial correlation tests (SPSS and Graphpad Prism packages; significant results for effect size higher than 0.4). Prognostic value of gDNA methylation and *rs1801133* variants considered survival profiles at 25 weeks of POH treatment, having the date of protocol adhesion as starting count and death as the final event.

**Results:**

Most rGBM patients showed global gDNA hypomethylation (median = 31.7%) and a significant, moderate and negative correlation between TT genotype and gDNA hypomethylation (median = 13.35%; rho = − 0.520; *p* = 0.003) compared to CC variant (median = 32.10%), which was not observed for CT variant (median = 33.34%; rho = − 0.289; *p* = 0.06). gDNA hypermethylated phenotype (median = 131.90%) exhibited significant, moderate and negative correlations between TT genotype (median = 112.02%) and gDNA hypermethylation levels when compared to CC (median = 132.45%; rho = − 0,450; *p* = 0.04) or CT (median = 137.80%; rho = − 0.518; *p* = 0.023) variants. TT variant of *rs1801133* significantly decreased gDNA methylation levels for both patient groups, when compared to CC (d values: hypomethylated = 1.189; hypermethylated = 0.979) or CT (d values: hypomethylated = 0.597; hypermethylated = 1.167) variants. Positive prognostic for rGBM patients may be assigned to gDNA hypermethylation for survivors above 25 weeks of treatment (median = 88 weeks); and TT variant of *rs1801133* regardless POH treatment length.

**Conclusion:**

rGBM patients under POH-based therapy harboring hypermethylated phenotype and TT variant for *rs1801133* had longer survival. Intranasal POH therapy mitigates detrimental effects of gDNA hypomethylation and improved survival of patients with rGBM harboring TT mutant variant for *MTHFR rs1801133* polymorphism.

**Trial registration:**

CONEP -9681- 25,000.009267 / 2004. Registered 12th July, 2004.

## Background

### DNA methylation status, folate metabolism and its relevance for glioblastoma patients under perillyl alcohol (POH) inhaled therapy

Similarities on global DNA methylation status among different tissues within the same individual indicate genetic influence on genomic DNA methylation status [1]. Genome-wide changes in DNA methylation pattern is a hallmark of cancer, particularly glioblastoma (GBM) [2]. GBM is the most common and highly proliferative brain tumor in adults. Genome-wide DNA hypomethylation in GBM supports an aggressive tumor phenotype by promoting genomic instability and oncogene activation, while loci-specific DNA hypermethylation silences tumor suppressor genes [3]. Epigenetic alteration in genomic DNA (gDNA) is a reversible and early event in malignant transformation, regulating gene expression and genomic stability [4]. Importantly, the reversible nature of gDNA methylation upon folate supplementation is being evaluated as a possible therapeutic strategy in animal models of glioma [3]. Nonetheless, prognosis of GBM patients remains dismal. Clinical presentation is often associated to functional damage caused by anatomical location of the tumor in the brain tissue; partly because tumor lesions may remain undetectable by magnetic resonance image for many years [5–8]. Despite recent advances on neuroimaging and therapeutic approaches, time to tumor recurrence in GBM patients treated with surgery followed by chemo-radiotherapy is only 25 to 40 weeks, with a median overall survival around 14 months after diagnosis [9–12].Therefore, following the emergence of drug-resistant clones, virtually all patients with GBM will suffer disease recurrence [13–15].

The naturally occurring monoterpene perillyl alcohol (POH) halts tumor progression and prolongs the overall survival in patients with treatment-refractory brain tumors [16–18]. Such encouraging findings result from the intranasal administration of POH, thus assuring the delivery of a pleiotropic compound with cytotoxic, pro-apoptotic, anti-inflammatory and antiangiogenic properties directly into the brain tissue. Moreover, delivery of POH through the intranasal route may avoid the hepatic first passage effect and maximize the direct antitumor effect of the treatment against malignant brain tumors [18–20].

Polymorphisms on the gene encoding the methylenetetrahydrofolate reductase (MTHFR) enzyme, which plays a key role in the folate-mediated one-carbon metabolism, also affects proliferation rates and disease progression, as tumor cells frequently rely on the folate metabolism as a major source of one-carbon units for anabolic processes, nucleic acid synthesis and DNA methylation [13, 21–26]. Functional polymorphism *rs1801133* (C677T) in the *MTHFR* gene consists of a cytosine (C) to thymidine (T) transition at the nucleotide 677, and results in an aminoacid change from alanine to valine at the encoded protein. Such change results in an enzyme with decreased activity due to its thermolabile property [27] and metabolic relevance both in the physiological highly anabolic brain parenchyma and in the highly metabolic demanding GBM context [28].

Recently, we demonstrated that levels of circulating molecular markers (cell-free DNA) reflect brain tumor activity [29] being a noninvasive screening tool for early tumor recurrence [30], prognostic evaluation and response to POH treatment [29].Since abnormalities on genomic DNA (gDNA) methylation is considered to be an early and reversible event of malignant transformation, the present study aimed to investigate the association between global gDNA methylation levels with functional polymorphism *rs1801133* (C677T) of the *MTHFR* gene and survival of patients with recurrent GBM (rGBM) undergoing POH-base therapy.

## Methods

### Study population

Before adhering to the protocol, all patients and/or his /her legal representative along with their primary care physician received detailed information about the study and agreed to participate in the Phase I/II study to evaluate the therapeutic efficacy of intranasal administration of the monoterpene perillyl alcohol (POH) by signing an informed consent form. As a criterion for inclusion in the POH protocol, a diagnosis of recurrent malignant GBM was required, based on the initial diagnosis from the histopathological and image findings, as well as the presence of detectable and contrasting-enhancing mass provided by magnetic resonance imaging (MRI) at the time of protocol inclusion. All patients included in this study had already failed all forms of conventional therapy (surgery, radiotherapy and specific chemotherapy for brain tumors) and were receiving symptomatic and palliative care. Retrospective data from one hundred adult patients with a proven diagnosis of recurrent GBM (rGBM) were considered by this study: 38 females, median age 50 years-old (range: 18 to 78 years) and 62 males, median age 52 years-old (range: 19 to 76 years).

POH was formulated to be used by inhalation by the University Pharmacy in accordance with international patent application US Patent Application 20,040,087,651 May 6, 2004 and Brazilian PI0107262–5 with approval by local Board of Health. The work followed the norms of the Helsinki Convention for clinical study and was approved by local research ethics committee (CONEP registration 9681 n^o^. 25000.009267 / 2004; CAAE0085.0.258.000–08).

### Extraction and quantification of genomic DNA

Extraction of genomic DNA obtained from the peripheral blood of rGBM patients followed the protocol described in the AxyPrep Blood Genomic DNA Miniprep kit (Axygen). Genomic DNA resulting from the extraction of whole blood was quantified by spectrophotometry (NanoVue Plus; GE Healthcare Life Sciences), and stored at − 20 °C until the moment of use.

### Global gDNA methylation levels

Global methylation levels were determined from 30 μL of DNA (200 ng) through the Imprint (Methylated DNA Quantification Kit, Sigma Chem Co., USA), following the manufacturer protocol. Since 70 to 90% of human genomic DNA obtained from somatic cells is considered to be methylated, and that DNA hypomethylation consists of a hallmark of malignant transformation [31–33] we established 90% as the experimental cut-off value for differentiation of both gDNA methylation phenotypes.

### Genotyping analysis

Genotyping of the 677C > T polymorphism in the MTHFR gene was performed by the PCR-RFLP (Restriction Fragment Length Polymorphism) method using the following primers: forward:

5′ GAAGCAGGGAGCTTTGAGGCTGACCT-3′ and reverse: 5′-AGGGATGCCCATGTCGGTGCATGCCT-3 ‘. Amplifications were performed using a Veriti® 96-well thermal cycler (Applied Biosystems, USA) under the following conditions: initial denaturation at 94 °C for 4 min, 35 cycles of 30 s at 94 °C, annealing at 65 °C for 30 s and extension at 72 °C for 30 s, followed by a final extension step of 72 °C for 5 min. For complete digestion of fragments, 5 μL of the PCR product was incubated at 65 °C for 3 h. A single fragment of 142 base pairs (bp) was identified as homozygous (CC) for the MTHFR normal allele; individuals bearing the 677 T polymorphic allele have a restriction site for the *TaqI* enzyme, which digests the 142 bp fragment at 58 and 84 bp and a three-fragment pattern of 142, 84, and 58 bp were identified as heterozygous genotype (CT) [34]. The restriction sites profile was visualized on 3% agarose gel using GelRed™ as a dye. For each digestion and enzymatic digestion verification carried out by electrophoresis, at least one previously confirmed polymorphic positive homozygote control was used.

### Statistical analysis

Mann-Whitney test was selected for comparison of two independent groups according to methylation phenotypes (hypomethylated or hypermethylated). Pearson Chi-square test was chosen to evaluate the Hardy-Weinberg (HWE) equilibrium and also to perform comparison of proportions of genotypes, followed by Bonferroni correction (significant results for adjusted *p* < 0.02). Kruskal-Wallis followed by pairwise comparison tests were employed to compare methylation median according to different genotypes of *rs1801133* (C677T), significant results for adjusted *p* < 0.02). Effect size estimation (d value) was performed in addition to multiple comparison tests to highlight the magnitude of the relationship of variables evaluated, when significant results were considered for d ≥ 0.4, related to the zone of desired effects) [35, 36]. Spearman point-biserial correlation was selected to evaluate the relationship between continuous and categorical variables (genotypes and global gDNA methylation levels) [37].Prognostic value of both gDNA methylation phenotypes and *rs 1801133MTHFR* variants was performed considering 25 weeks (cut-off) as critical reference time for POH treatment evaluation, considering the historical survival data of rGBM patients that fail response to standard therapeutic scheme [12]. For such analysis, the date of protocol adhesion was considered as the starting point and the date of death as final event (*n* = 67). Patients who abandoned the study protocol were not considered for such evaluation (*n* = 33). All the above-mentioned statistical tests were run at SPSS 20.0 and GraphPad Prism 5.0 softwares.

## Results

### rGBM patients with hypomethylated gDNA and worse prognosis during POH inhaled therapy

**Fig. 1:**
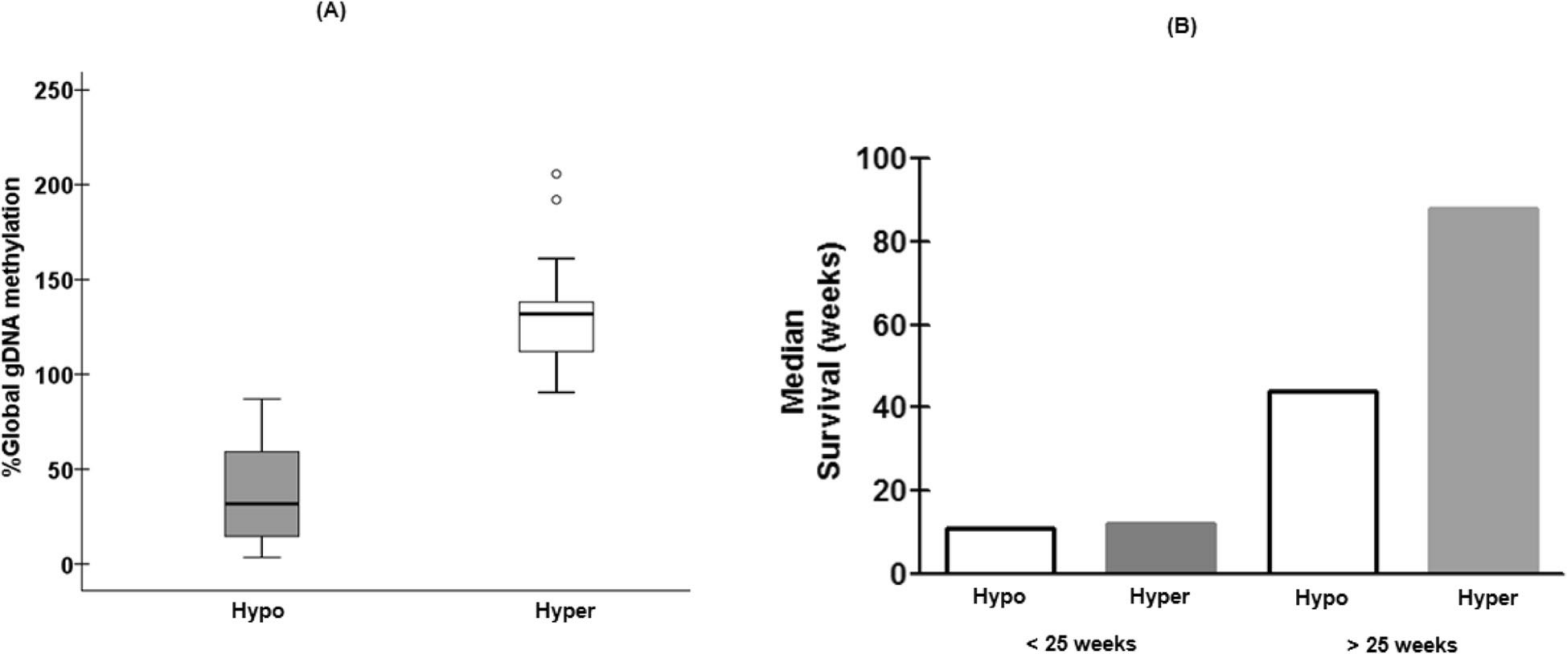
**a** Global gDNA methylation pattern and prognosis of rGBM patients under POH inhaled therapy. The majority of rGBM patients (67%) presented with hypomethylated phenotype compared to hypermethylated group (33%, *p* < 0.0001). **b** Multiple comparison tests at 25 weeks confirm survival advantage to gDNA hypermethylated patients (median survival = 88 weeks) in comparison to hypomethylated rGBM survivors above 25 weeks (median survival = 44 weeks; d = 0.639)

Global DNA methylation status of rGBM patients were determined using gDNA extracted from peripheral blood mononuclear cells. Patients were assigned to the hypomethylated group if individual global gDNA methylation levels were ≤ 90%; and to the hypermethylated group if gDNA methylation levels were > 90% [31]. The majority (67%) of the rGBM patients presented with hypomethylated gDNA (median = 31.7%; range: 3.4 to 87.0%) vs. the hypermethylated group (33%; median = 131.9%; range: 90.4 to 206%), with a significant difference (*p* < 0.0001; d = 2.766) between both groups (Fig. 1a).

Since gDNA methylation abnormalities are a hallmark of tumor progression [2], we proceeded to evaluate the impact of gDNA methylation status on the overall survival of patients undergoing POH-based treatment considering 25 weeks as survival time cutoff value. Indeed, the multiple comparison tests performed at this specific time point for each distinct gDNA methylation phenotypes (Fig. 1b; Supplementary Table 1) showed significant differences between such groups (*p* < 0.0001; d = 3.234). Pair wise comparisons showed significant differences (d = 0.639) in terms of median survival times between gDNA hypomethylated (44 weeks) and hypermethylated phenotypes (88 weeks) for those rGBM patients with survival times higher than 25 weeks. Conversely, rGBM patients who survived less than 25 weeks did not present median survival differences (*p* = 0.811; d = 0.093) despite of significant distinct gDNA methylation profiles (41.40% vs 135.98%; *p* < 0.0001, d = 2.941).Significant differences were observed for median survival profiles within hypomethylated (< 25 weeks = 11 weeks; > 25 weeks = 44 weeks; *p* < 0.0001, d = 2.991) and within hypermethylated (< 25 weeks = 12 weeks; > 25 weeks = 88 weeks; *p* < 0.0001, d = 3.00) groups. Finally, median survival profiles above or below 25 weeks were significantly different according both gDNA methylation phenotypes. Such results indicate longer survival times for rGBM survivors above 25 weeks, despite significant differences on gDNA methylation profiles.

### The *rs1801133* (C677T) *MTHFR* TT variant is associated with lower gDNA methylation levels

Given the role of *MTHFR* C677T polymorphism to sustain anabolic pathways in rapidly proliferating tumor cells, we proceeded to evaluate the frequency of rGBM patients harboring different *rs1801133* (C677T) genotypes, and to assess whether individual *MTHFR* C677T variants correlate with both gDNA methylation patterns. Genotype analysis of rGBM patients showed a compliance with HWE (*p* = 0.65). We observed homozygous CC genotype in 38% of rGBM patients, while 49% of the patients carry the heterozygous CT genotype, and the remaining 13% carry the TT genotype. The comparison of different proportions of each genotype showed significant differences between observed frequencies for genotypes CC and TT (*p* = 0.003), and CT and TT (*p* < 0.0001).

**Fig. 2:**
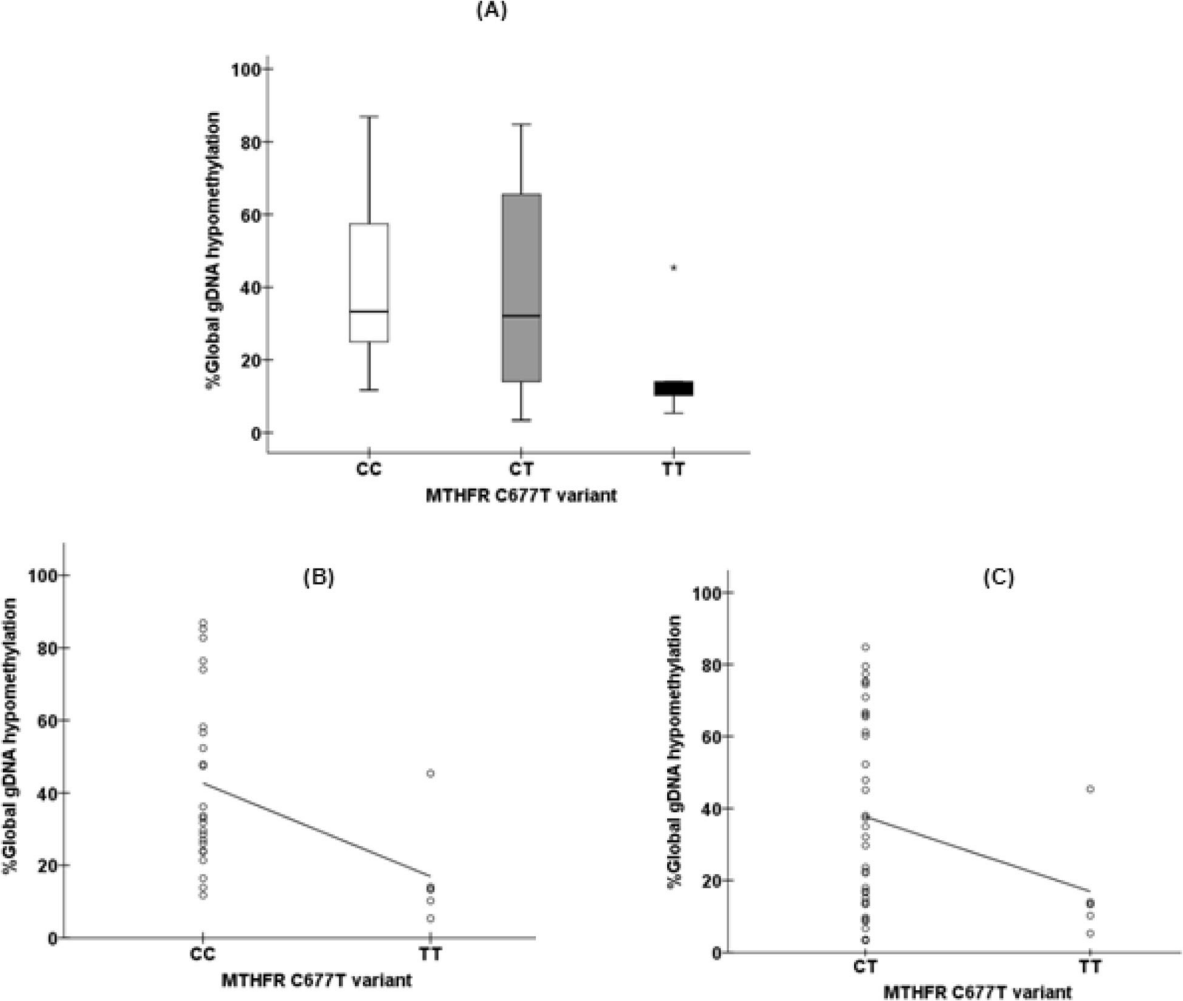
**a** Multiple comparison of gDNA hypomethylation median levels according *rs1801133* variants (*p* = 0.033, d = 0.570) show that rGBM patients harboring the TT genotype variant presented with a significant and marked reduction on gDNA hypomethylation (median = 13.35%) with a 2.4-fold decrease when compared to CC (median = 33.34%, d = 1.189) and CT (median = 32.10%, d = 0.597) genotypes. We further observed a significant, moderate and negative correlation between TT or CC (rho = − 0.520; *p* = 0.003, figure 2**b**) genotypes and a non-significant, weak and negative correlation between TT or CT (rho = − 0.289; *p* = 0.06, figure 2**c**) variants and global gDNA hypomethylation levels

Multiple comparison test showed significant differences between gDNA hypomethylation median results according each *MTHFR* genetic variant (d value of Kruskal-Wallis test = 0.570, Fig. 2a, Supplementary Table 2). Compared to *rs1801133* CC genotype, rGBM patients with the TT genotype presented with a 2.4-fold lower level of gDNA hypomethylation [median = 13.35% (range: 5.32 to 45.4%) vs. median = 33.34% (range 11.7 to 87%), respectively; *p* = 0.005, d = 1.189, Fig. 2a, Supplementary Table 2)]. The same magnitude (2.4-fold) upon gDNA hypomethylation reduction was observed between TT and CT *rs1801133* variants [median = 13.35% (range: 5.32 to 45.4%) vs. median = 32.10% (range 3.4 to 84.8%)], respectively; d = 0.597, Fig. 2a, Supplementary Table 2)]. In addition, we observed a significant, moderate and negative correlation between the TT genotype and gDNA hypomethylation compared to CC (rho = − 0.520; *p* = 0.003, Fig. 2b) but a non-significant, weak and negative correlation between TT variant and gDNA hypomethylation (rho = − 0.289; *p* = 0.06, Fig. 2c) when compared tothe CT variant.

**Fig. 3:**
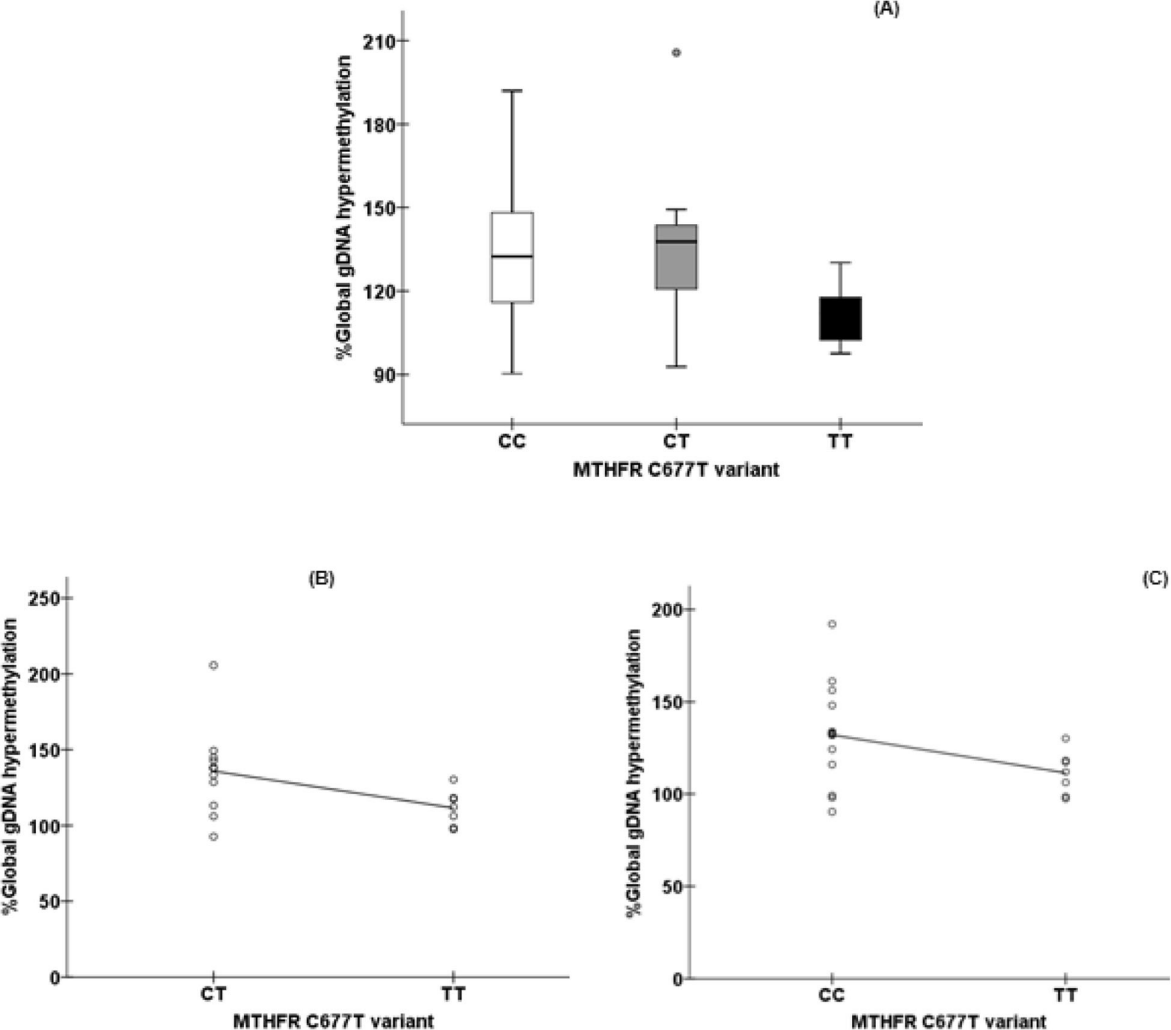
**a** Multiple comparison of gDNA hypermethylation median levels according *rs1801133* variants (d = 0.751) show that rGBM patients harboring the TT genotype variant presented a significant and marked reduction (1.2-fold) on gDNA hypermethylation (median = 112.02%) when compared to CC (median = 132.45%, d = 0.979) and CT (median = 137.80%,d = 1.167) genotypes. Significant, moderate and negative correlations between TT or CC (rho = − 0.450; *p* = 0.040, figure 3**b**) genotypes, and TT or CT (rho = − 0.518; *p* = 0.023, figure 3**c**) variants and global gDNA hypermethylation levels

Regarding hypermethylated phenotype, multiple comparison test between *rs1801133* variants showed significant differences on median gDNA methylation levels (d value for Kruskal-Wallis test =0.751, Fig. 3a, Supplementary Table 3). gDNA hypermethylation profile from individuals carrying the *rs1801133* TT variant showed a significant 1.2-fold decrease in hypermethylation levels [median = 112.02% [range: 97.5 to 130.20%] compared to CC genotype [median = 132.45% [range 90.4 to 192.03%]; d = 0.979, Fig. 3a] and to CT genotype [median = 137.80% [range 92.7 to 206%]; d = 1.167, Fig. 3a]. In addition, we further observed significant, moderate and negative correlations between the TT genotype and gDNA hypermethylation compared to CC (rho = − 0.450; *p* = 0.040; Fig. 3b) and CT (rho = − 0.518; *p* = 0.023; Fig. 3c) genotypes.

### Survival analysis of rGBM patients under POH therapy according to different rs1801133 (C677T) genotypes

**Fig. 4:**
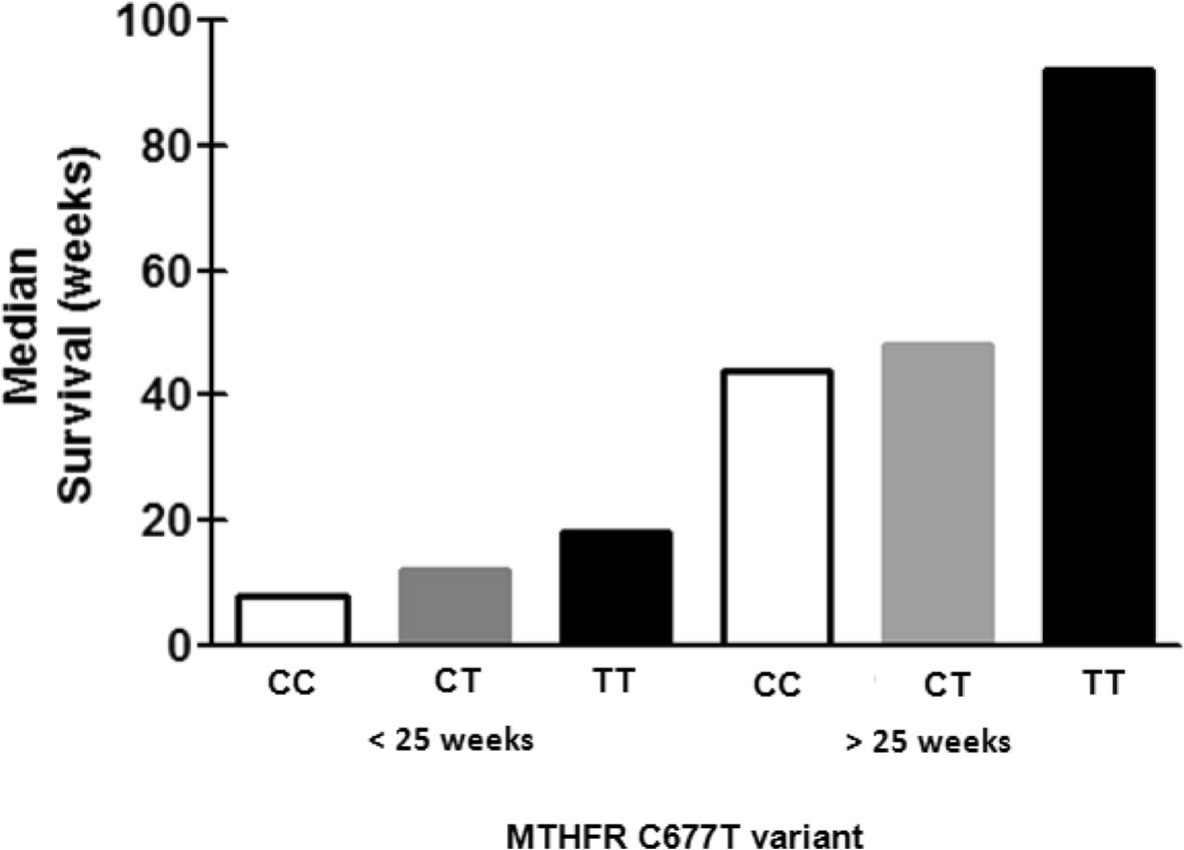
Multiple comparison tests at 25 weeks confirm positive prognosis to rGBM patients harboring the TT polymorphic variant of *MTHFR rs1801133* in comparison to the remaining variants, both for patients with lower survival times (TT = 18 weeks; CC = 8 weeks; d = 1.583 and CT = 12 weeks, d = 0.941), as well as for those who achieved higher survival times (TT = 92 weeks; CC = 44 weeks; d = 0.648 and CT = 48 weeks, d = 0.807). Besides, significant prognosis differences were also observed within and between such *MTHFR* variants after and before the specific moment during inhaled POH treatment (Supplementary Table 4)

Taking into account survival differences between both gDNA methylation groups at a specific time during treatment course (25 weeks, Fig. 1), and the impact of *rs1801133* (C677T) polymorphic variants on gDNA methylation levels (Figs. 2 and 3), we considered relevant to evaluate the relationship between these polymorphic variants and survival at 25 weeks for our rGBM cohort. Indeed, multiple comparison tests showed significant differences between MTHFR *rs1801133* variants for those rGBM survivors who lived more or less than 25 weeks (*p* < 0.0001; d = 3.247). Pair wise comparison showed that rGBM patients with TT variant had better prognosis (18 weeks) when compared to CC or CT variants for those patients with lower survival (CC = 8 weeks; d = 1.583 and CT = 12 weeks, d = 0.941) and for those with higher survival (TT = 92 weeks; CC = 44 weeks; d = 0.648 and CT = 48 weeks, d = 0.807). It was also observed significant survival differences within and between *MTHFR rs1801133* variants after and before the selected median time (25 weeks) of survival after tumor recurrence (Supplementary Table 4, Fig. 4).

## Discussion

Currently available treatments for rGBM are unable to effectively overcome the high intratumoral cellular and molecular heterogeneity to elicit sustained tumor regression. Disease complexity may be further enhanced by detrimental effects of cytotoxic chemotherapy and ionizing radiation on genetic and epigenetic stability [15, 22, 26].Since tumor-associated changes in the gDNA methylation status is replicated in gDNA obtained from peripheral blood cells of cancer patients [38–40], analysis of gDNA global methylation status using peripheral mononuclear cells from rGBM patients could be potentially used as a non-invasive prognostic biomarker, representing a valuable circulating screening tool that better represents the dynamic nature of such intracranial malignancy [41].

Besides, the brain parenchyma under physiological conditions, presents a highly anabolic metabolism, where one-carbon units consist of key elements for macromolecules synthesis, gene expression control and redox balance [28]. The recognized impact of *rs1801133* (C677T) functional polymorphism impairment on the key regulatory enzyme (MTHFR) of folate metabolism gains increased relevance in the context of highly invasive and proliferative glioblastoma, especially if considered the tumor cells metabolic reprogramming as a key pillar of malignant transformation [42] [43, 44].

In the present study, we used gDNA isolated from peripheral blood samples to examine whether global gDNA methylation status and more specifically, *rs1801133* polymorphism of the *MTHFR* gene, correlated with survival outcomes of rGBM patients undergoing intranasal POH treatment. All rGBM patients enrolled in this study were at terminal stage, after failing response to all therapeutic schemes (standard and second line therapies) and just receiving support and palliative care. In agreement with previous reports, we observed that the majority of rGBM patients presented wide gDNA hypomethylation [45]. Despite the significant survival differences for both methylation groups (44 weeks for hypomethylated vs 88 weeks for hypermethylated gDNA) after a specific moment of treatment (25 weeks), the present study corroborates previous data from our group [16–18], strongly reinforcing the efficacy of POH inhaled therapy on prolonging rGBM patients survival for both methylation groups, achieving substantial increment for both hypo (76%) and hypermethylated phenotypes (252%).

The global DNA methylation status is influenced by several factors, including dietary intake of enzymatic cofactors such as B9 (folate), B12 and B6 vitamins. Such cofactors play a key role in MTHFR-mediated one-carbon metabolic pathway, thus impacting overall DNA methylation levels [46]. Genotype analysis of our rGBM patients’ cohort showed that 62% of patients had at least one mutated allele for the *rs1801133MTHFR* gene polymorphism (CT = 49%; TT = 13%). Important worth mentioning that reports on the role of *MTHFR* polymorphism in GBM were often case-control studies, mainly investigating the influence of genotypic variants on disease risk [47–49], which clearly was not the scope of the present study. Nevertheless, we are aware that caution should be taken when making comparisons between genotype frequencies among different patient populations, being imperative to consider the influence that ancestry and population heterogeneity play in each particular study.

Regardless the wide distribution of global gDNA methylation status among rGBM patients, we identified significant and negative correlations of this epigenetic signature between rGBM patients harboring the TT genotype compared to homozygous (CC genotype) variant (Figs. 2 and 3). Indeed, comparing to CC genotype the TT genotype showed significant reduction in gDNA methylation levels in both the hypomethylated group (2.4-fold decrease; d = 1.189; Supplementary Table 2) and the hypermethylated group (1.2-fold decrease; d = 0.979, Supplementary Table 3). Interestingly, even for the hypermethylated phenotype, the homozygous TT polymorphic variant showed an expressive change in global gDNA methylation levels, being more prominent in the hypomethylated rGBM patients. Such result emphasizes the importance of the proper functionality of the key regulatory MTHFR enzyme on global DNA methylation levels.

The decrease at global DNA methylation levels observed among rGBM patients with TT genotype would suggest worse prognosis for such patients, since this mutated genotype is associated to a drastic MTHFR loss of enzymatic functionality, impacting on decreasing the global DNA methylation scores, which is described as a marker of genesis and tumor progression [3, 4, 50, 51]. Surprisingly, when considered the same survival time (25 weeks) from which there were observed significant differences (d = 0.639) between the rGBM patients survival according the phenotypes hypo (44 weeks) and hypermethylated (88 weeks; Fig. 2b, Supplementary Table 1), it was possible to identify better survival times for individuals harboring the TT *rs1801133* genetic variant, despite methylation level (Supplementary Table 3). It is worth to mention the multifactorial essence of glioblastoma prognosis considering age at onset, tumor location, histology and molecular characteristics [52]. Despite of the lack of an appropriate sample size to run predictive models to study our rGBM cohort prognosis as a limitation of our study, as well as the recognized the intratumoral heterogeneity, the present results strongly demonstrates the role of gDNA global methylation and *rs1801133 MTHFR* variants as significant individual epigenetic and genetic candidates for rGBM patients under POH inhaled therapy prognosis. Besides, it seems plausible to hypothesize that due its pleiotropic nature, the monoterpene POH mitigates the overall impact of enzymatic MTHFR deficiency. This effect could be a result of POH-mediated indirect effects over additional metabolic and signaling pathways [53] that combined enhanced patients’ survival regardless of individual gDNA methylation status. Hence, even though decreased MTHFR activity tend to increase DNA synthesis and support rapid tumor cell proliferation, POH inhaled treatment could partially overcome this enzymatic deficiency promoting cell cycle arrest, apoptosis and inhibiting ROS harmful effects [19].

## Conclusion

The confirmation of the hypomethylated phenotype in rGBM patients using peripheral blood cells as biological source of gDNA confirms the utility of such non-invasive approach for our patients’ follow-up, since longer survival times were achieved by inhaled POH therapy. When compared to wild CC genotype, the homozygous TT genotype of *rs1801133* (C677T) of *MTHFR* gene may be associated with an overall decrease in global gDNA methylation and longer survival time of rGBM patients under POH-based therapy. Altogether, our results suggest that inhalation treatment of rGBM patients with POH mitigate the detrimental effects of global gDNA hypomethylation on tumor cell proliferation while fostering normalization of the MTHFR-mediate one-carbon folate metabolism in patients with recurrent glioblastoma harboring mutant variants for *rs1801133* polymorphism. Importantly, baseline characteristics of the patient cohort showing a significant benefit from inhaled POH therapy (> 25 weeks survivors) can introduce unknown bias into the results; therefore, additional studies may be required to further validate the findings reported in this study.

## Supplementary information

**Additional file 1: Table S1.** Comparison between Hypo and Hypermethylated patients at 25 weeks of POH treatment. **Table S2.** gDNA hypomethylation levels according *rs1801133* (C677T) *MTHFR* variants. **Table S3:** gDNA hypermethylation levels according *rs1801133* (C677T) *MTHFR* variants. **Table S4.** Prognostic relevance of *rs1801133* variants of rGBM patients at 25 weeks of POH treatment.

## Data Availability

The data generated and analyzed during the current study are not publicly available due confidentiality requirements, but are available from the corresponding author on reasonable request.

## Abbreviations

GBM: Glioblastoma
gDNA: Genomic DNA
HWE: Hardy-Weinberg equilibrium
MTHFR: Methylenetetrahydrofolate reductase
POH: Perillyl alcohol
rGBM: Recurrent glioblastoma
rs1801131: Reference Single nucleotide polymorphism number 1801131
