# Supplementary material for: Intranasal perillyl alcohol therapy improves survival of patients with recurrent glioblastoma harboring mutant variant for MTHFR rs1801133 polymorphism
Source: BMC Cancer. 2020 Apr 7;20:294. doi: 10.1186/s12885-020-06802-8 (PMC7137265; doi:10.1186/s12885-020-06802-8)
Supplement: Supplementary file 1 — Additional file 1: Table S1. Comparison between Hypo and Hypermethylated patients at 25 weeks of POH treatment. Table S2. gDNA hypomethylation levels according rs1801133 (C677T) MTHFR variants. Table S3: gDNA hypermethylation levels according rs1801133 (C677T) MTHFR variants. Table S4. Prognostic relevance of rs1801133 variants of rGBM patients at 25 weeks of POH treatment. [file 12885_2020_6802_MOESM1_ESM.docx]

**Supplementary Table 1: Comparison between Hypo and Hypermethylated patients at 25 weeks of POH treatment**

|  | **Survival (weeks)** | | | **% gDNA global methylation** | | |
| --- | --- | --- | --- | --- | --- | --- |
| **gDNA methylation groups** | **Median** | **p** | **d** | **Median (range)** | **p** | **d** |
| Hypo > 25 wks | 44 (28 - 176) | 0.051 | 0.639 | 25.35 (3.4 – 87) | <0.0001 | 2.912 |
| Hyper > 25 wks | 88 (28 - 504) |  |  | 117.27 (90.4 – 192.03) |  |  |
| Hypo <25 wks | 11 (2 - 24) | 0.811 | 0.093 | 41.40 (10.20 – 84.80) | <0.0001 | 2.941 |
| Hyper < 25 wks | 12 (4 - 20) |  |  | 135.98 (92.7 – 206) |  |  |
| Hypo < 25 wks | 11 (2 -24) | <0.0001 | 2.991 | 41.40 (10.20 – 84.80) | 0.123 | 0.49 |
| Hypo > 25 wks | 44 (28 - 176) |  |  | 25.35 (3.4 – 87) |  |  |
| Hyper < 25 wks | 12 (4 - 20) | <0.0001 | 3.00 | 135.98 (92.7 – 206) | 0.166 | 0.577 |
| Hyper > 25 wks | 88 (28 - 504) |  |  | 117.27 (90.4 – 192.03) |  |  |
| Hypo < 25 wks | 11 (2 - 24) | <0.0001 | 3.254 | 41.40 (10.20 – 84.80) | <0.0001 | 3.254 |
| Hyper > 25 wks | 88 (28 - 504) |  |  | 117.27 (90.4 – 192.03) |  |  |
| Hypo > 25 wks | 44 (28 - 176) | <0.0001 | 2.377 | 25.35 (3.4 – 87) | <0.0001 | 2.377 |
| Hyper < 25 wks | 12 (4 - 20) |  |  | 135.98 (92.7 – 206) |  |  |

Effect size (d): large = 0.80; medium = 0.50; small = 0.20. Adjusted p value for multiple comparisons was considered significant at 0.008 level

(Kruskal-Wallis survival test: p<0.0001; d=3.234; Kruskal-Wallis gDNA methylation test: p<0.0001; d=3.107).

**Supplementary table 2: gDNA hypomethylation levels according *rs1801133* (C677T) *MTHFR* variants**

| ***MTHFR* Genotype** | **gDNA Hypomethylation (%)** | | |
| --- | --- | --- | --- |
|  | **Median (range)** | **p** | **d** |
| CC | 33.34 (11.70 – 87) | 0.033 | 0.570 |
| CT | 32.10 (3.40 – 84.80) |  |  |
| TT | 13.35 (5.32 – 45.40) |  |  |
| CC | 33.34 (11.70 – 87) | 0.005 | 1.189 |
| TT | 13.35 (5.32 – 45.40) |  |  |
| CC | 33.34 (11.70 – 87) | 0.295 | 0.271 |
| CT | 32.10 (3.40 – 84.80) |  |  |
| CT | 32.10 (3.40 – 84.80) | 0.061 | 0.597 |
| TT | 13.35 (5.32 – 45.40) |  |  |

Effect size (d): large = 0.80; medium = 0.50; small = 0.20. Adjusted p value for multiple comparisons was considered significant at 0.017 level (p<0.017).

**Supplementary Table 3: gDNA hypermethylation levels according *rs1801133* (C677T) *MTHFR* variants**

| ***MTHFR* Genotype** | **gDNA Hypomethylation (%)** | | |
| --- | --- | --- | --- |
|  | **Median (range)** | **p** | **d** |
| CC | 132.45 (90.4 – 192.03) | 0.058 | 0.751 |
| CT | 137.80 (92.70 – 206) |  |  |
| TT | 112.02 (97.50 – 130.20) |  |  |
| CC | 132.45 (90.4 – 192.03) | 0.044 | 0.979 |
| TT | 112.02 (97.50 – 130.20) |  |  |
| CC | 132.45 (90.4 – 192.03) | 0.572 | 0.223 |
| CT | 137.80 (92.70 – 206) |  |  |
| CT | 137.80 (92.70 – 206) | 0.028 | 1.167 |
| TT | 112.02 (97.50 – 130.20) |  |  |

Effect size (d): large = 0.80; medium = 0.50; small = 0.20. Adjusted p value for multiple comparisons was considered significant at 0.017 level (p<0.017).

**Supplementary table 4: Prognostic relevance of *rs1801133* variants of rGBM patients at 25 weeks of POH treatment**

|  | **Survival (weeks)** | | |
| --- | --- | --- | --- |
| ***rs1801133* MTHFR variant** | **Median** | **p** | **d** |
| CC > 25 weeks | 44 (28 – 208) | 0.147 | 0.648 |
| TT > 25 weeks | 92 (28 – 504) |  |  |
| CT > 25 weeks | 48 (28 – 176) | 0.056 | 0.807 |
| TT > 25 weeks | 92 (28 – 504) |  |  |
| CC > 25 weeks | 44 (28 – 208) | 0.917 | 0.036 |
| CT > 25 weeks | 48 (28 – 176) |  |  |
| CC < 25 weeks | 8 (2 – 24) | 0.023 | 1.583 |
| TT < 25 weeks | 18 (17 – 24) |  |  |
| CT < 25 weeks | 12 (2 – 24) | 0.077 | 0.941 |
| TT < 25 weeks | 18 (17 – 24) |  |  |
| CC < 25 weeks | 8 (2 – 24) | 0.459 | 0.017 |
| CT < 25 weeks | 12 (2 – 24) |  |  |
| CC > 25 weeks | 44 (28 – 208) | < 0.0001 | 2.882 |
| CC < 25 weeks | 8 (2 – 24) |  |  |
| CT > 25 weeks | 48 (28 – 176) | <0.0001 | 3.167 |
| CT < 25 weeks | 12 (2 – 24) |  |  |
| TT > 25 weeks | 92 (28 – 504) | 0.008 | 2.646 |
| TT < 25 weeks | 18 (17 – 24) |  |  |
| CC > 25 weeks | 44 (28 – 208) | < 0.0001 | 3.211 |
| CT < 25 weeks | 12 (2 – 24) |  |  |
| CC > 25 weeks | 44 (28 – 208) | 0.003 | 1.897 |
| TT < 25 weeks | 18 (17 – 24) |  |  |
| CC < 25 weeks | 8 (2 – 24) | < 0.0001 | 2.62 |
| CT > 25 weeks | 48 (28 – 176) |  |  |
| CC < 25 weeks | 8 (2 – 24) | 0.001 | 3.018 |
| TT > 25 weeks | 92 (28 – 504) |  |  |
| CT > 25 weeks | 48 (28 – 176) | 0.002 | 1.678 |
| TT < 25 weeks | 18 (17 – 24) |  |  |
| CT < 25 weeks | 12 (2 – 24) | < 0.0001 | 2.726 |
| TT > 25 weeks | 92 (28 – 504) |  |  |

Effect size (d): large = 0.80; medium = 0.50; small = 0.20. Adjusted p value for multiple comparisons was considered significant at 0.003 level

(Kruskal-Wallis test: p<0.0001; d=3.247).
